# Supplementary material for: The Microbiome Structure of a Rice-Crayfish Integrated Breeding Model and Its Association with Crayfish Growth and Water Quality
Source: Microbiol Spectr. 2022 Apr 6;10(2):e02204-21. doi: 10.1128/spectrum.02204-21 (PMC9045173; doi:10.1128/spectrum.02204-21)
Supplement: SUPPLEMENTAL FILE 1 — Supplemental material. Download SPECTRUM02204-21_Supp_1_seq12.pdf, PDF file, 2.0 MB [file spectrum02204-21_supp_1_seq12.pdf]

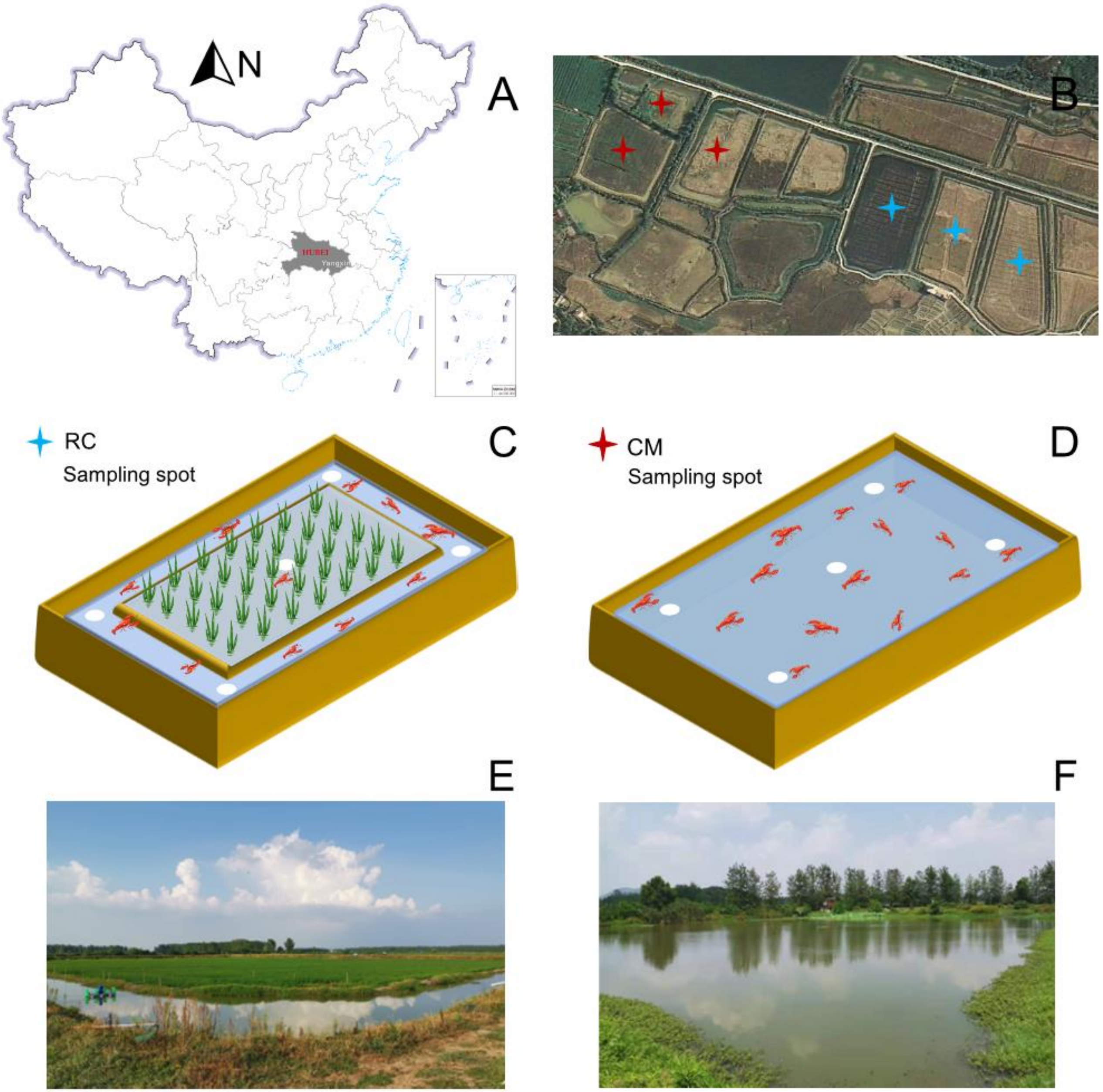

Fig S1. The sampling location and overall view of two experimental fields of crayfish cultivation. A, The geographical location of sampling sites. B, The satellite images of sampling sites for the RC and CM models. C, The sampling spots of the RC model. D, The sampling spots of the CM model. E, The overview of the RC experimental field. F, The overview of the CM experimental field.

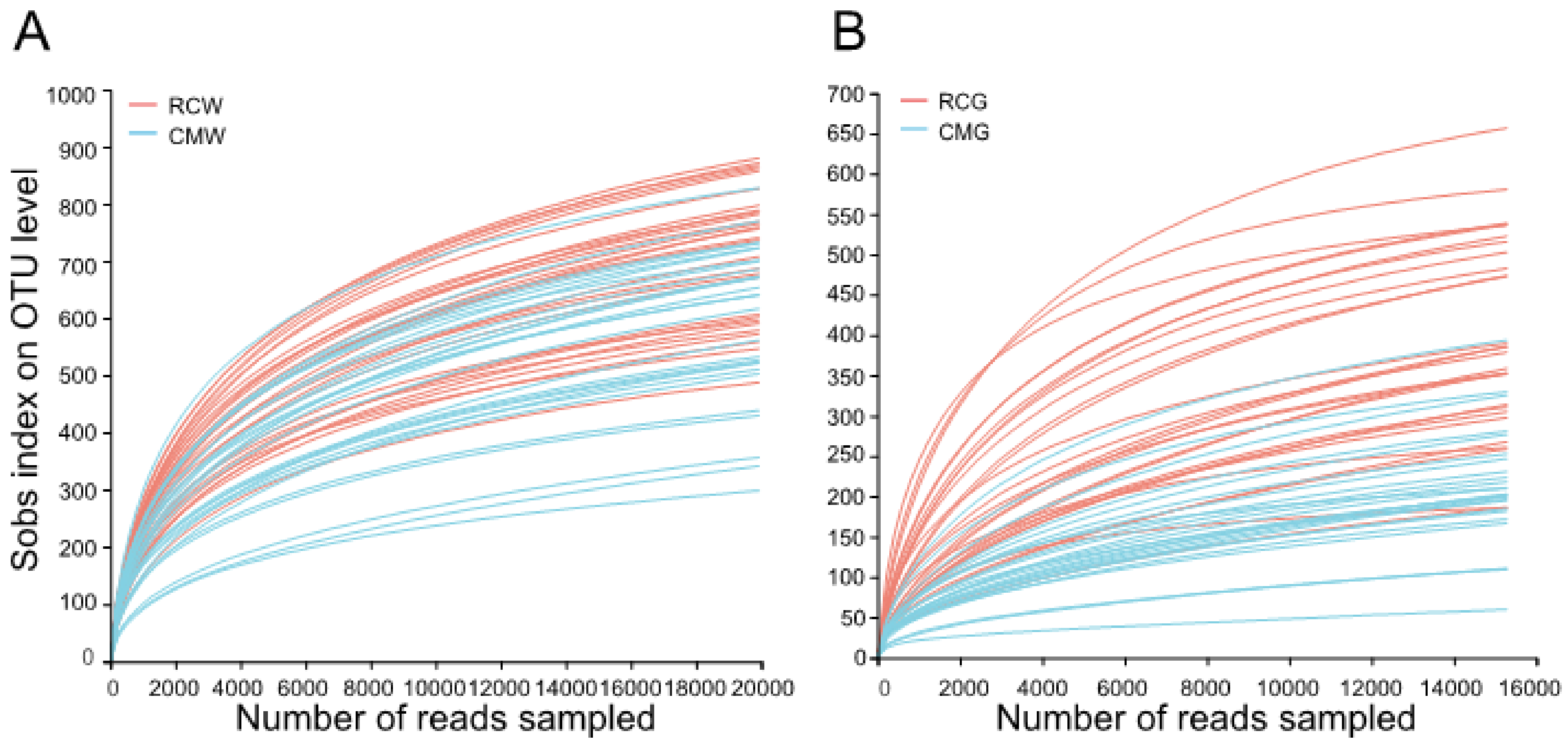

Fig S2. Rarefaction curves of 16S rDNA amplicon sequencing depth in water (A) and gut (B) microbiomes.

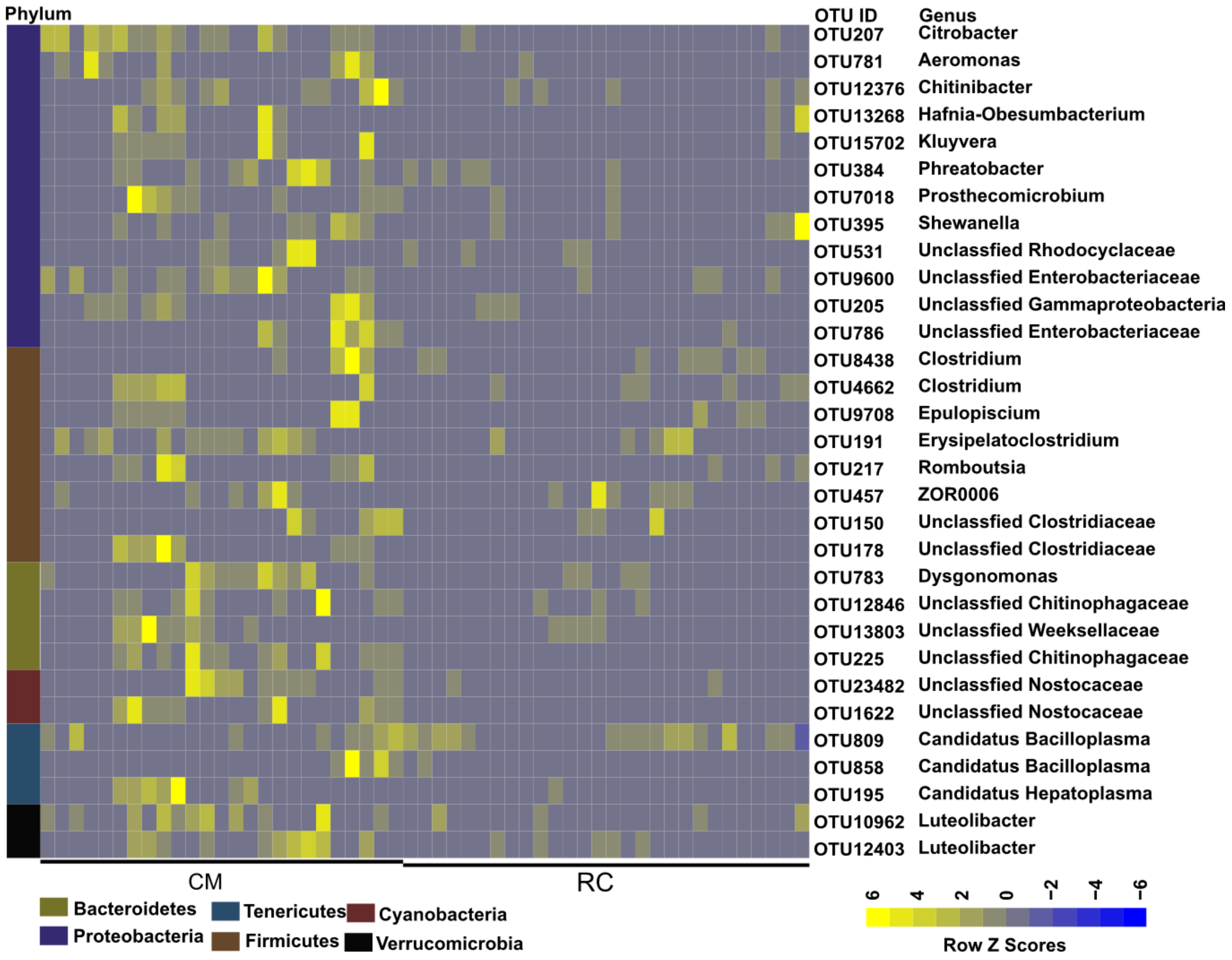

Fig S3. Relative abundances of the RC-depleted gut microbiome at OTU level. Scale, relative abundance of OTU at row normalization by removing the mean (centering) and dividing by the standard deviation (scaling). The color from blue to yellow represents a relative abundance of each taxon from low to high.

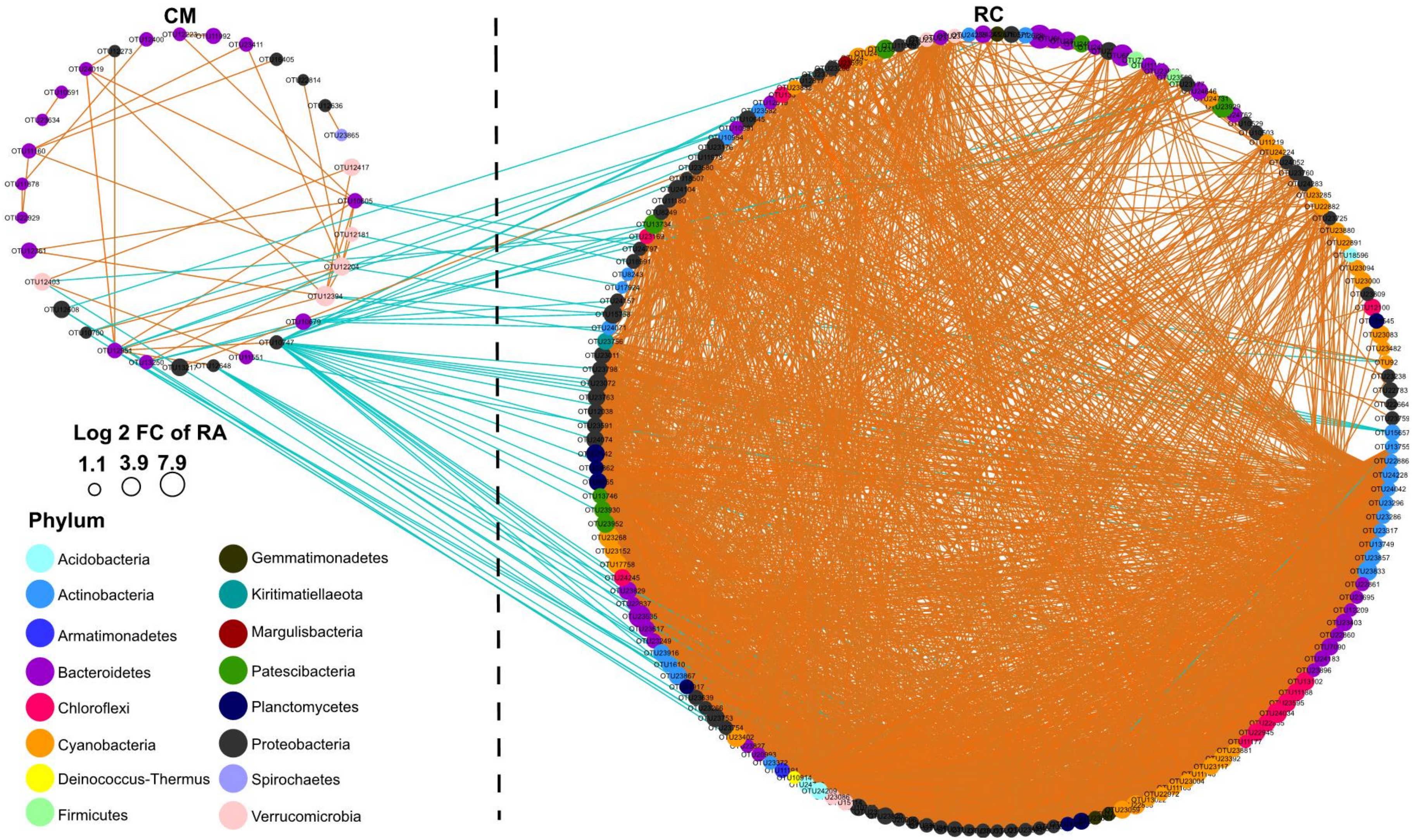

Fig S4. The co-occurrence patterns among the RC-CM niche-differentiated OTUs of water microbiome. The nodes were colored according to their taxonomic annotation, and the node size denotes the $\log_2$ fold change (FC) of relative abundance (RA) of the OTUs between the RC and CM models. Left of dish line means the RC-depleted OTUs, and OTUs on the right of dish line are the RC-enriched OTUs.

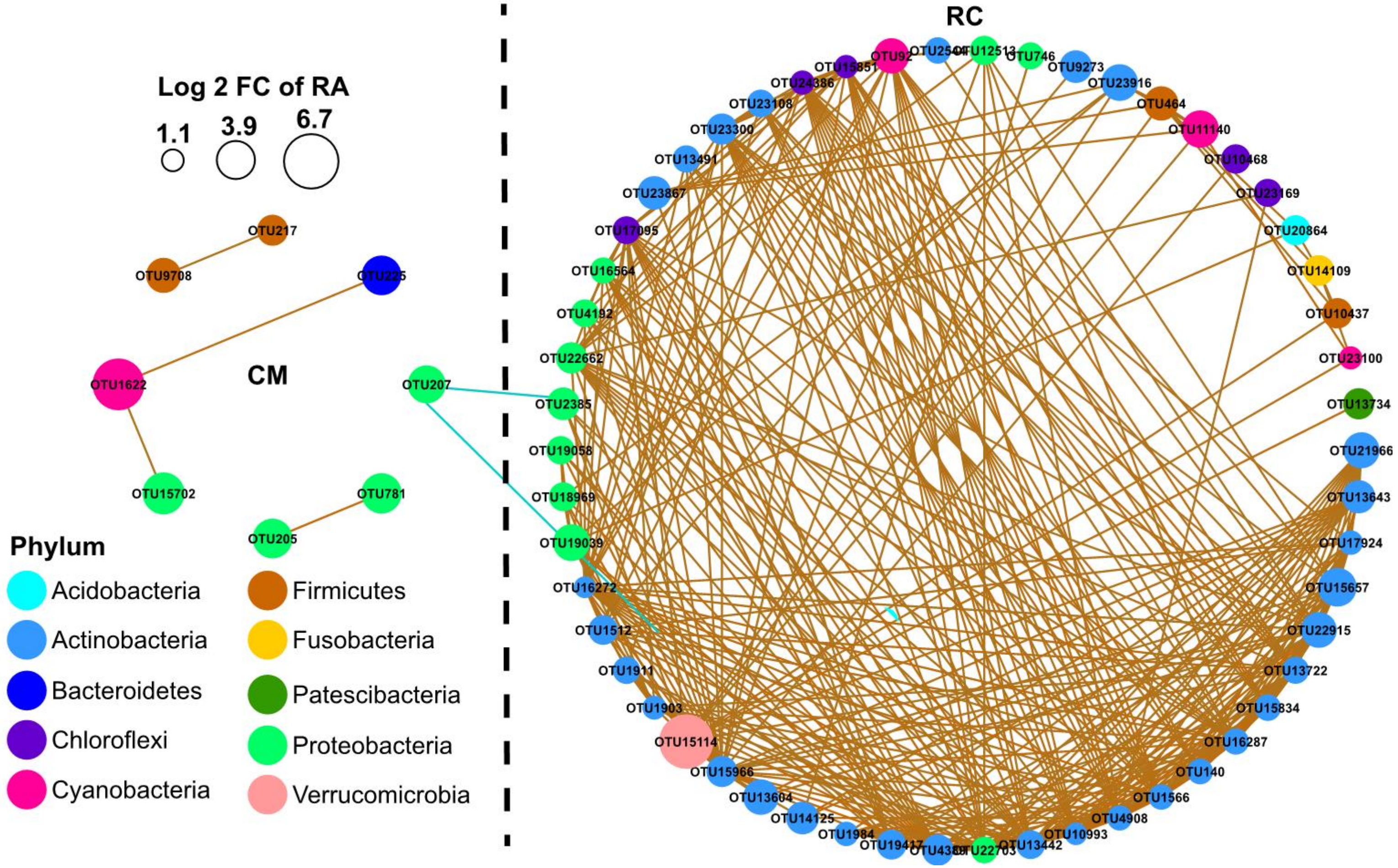

Fig S5. The co-occurrence patterns among the RC-CM niche-differentiated OTUs of gut microbiome. The nodes were colored according to their taxonomic annotation, and the node size denotes the log2 fold change (FC) of relative abundance (RA) of the OTUs between the RC and CM models. Left of dish line, the RC-depleted OTUs, and OTUs on the right of dish line are the RC-enriched OTUs.

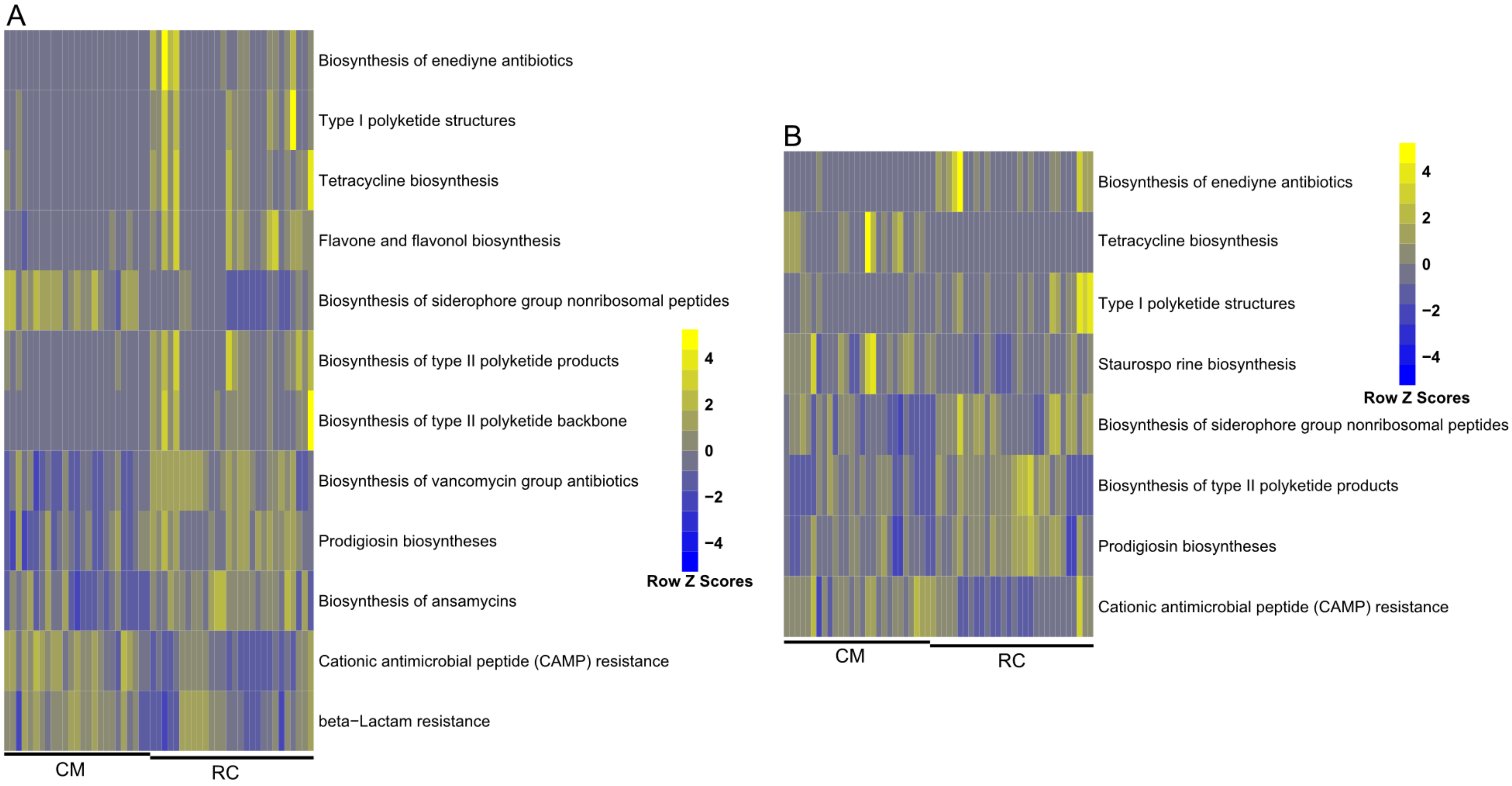

Fig S6. Relative abundance comparisons of KEGG level3 pathway in gut (A) and water (B) microbiomes between the RC and CM models. Scale, relative abundance of OTU at row normalization by removing the mean (centering) and dividing by the standard deviation (scaling). The color from blue to yellow represents a relative abundance of each taxon from low to high.

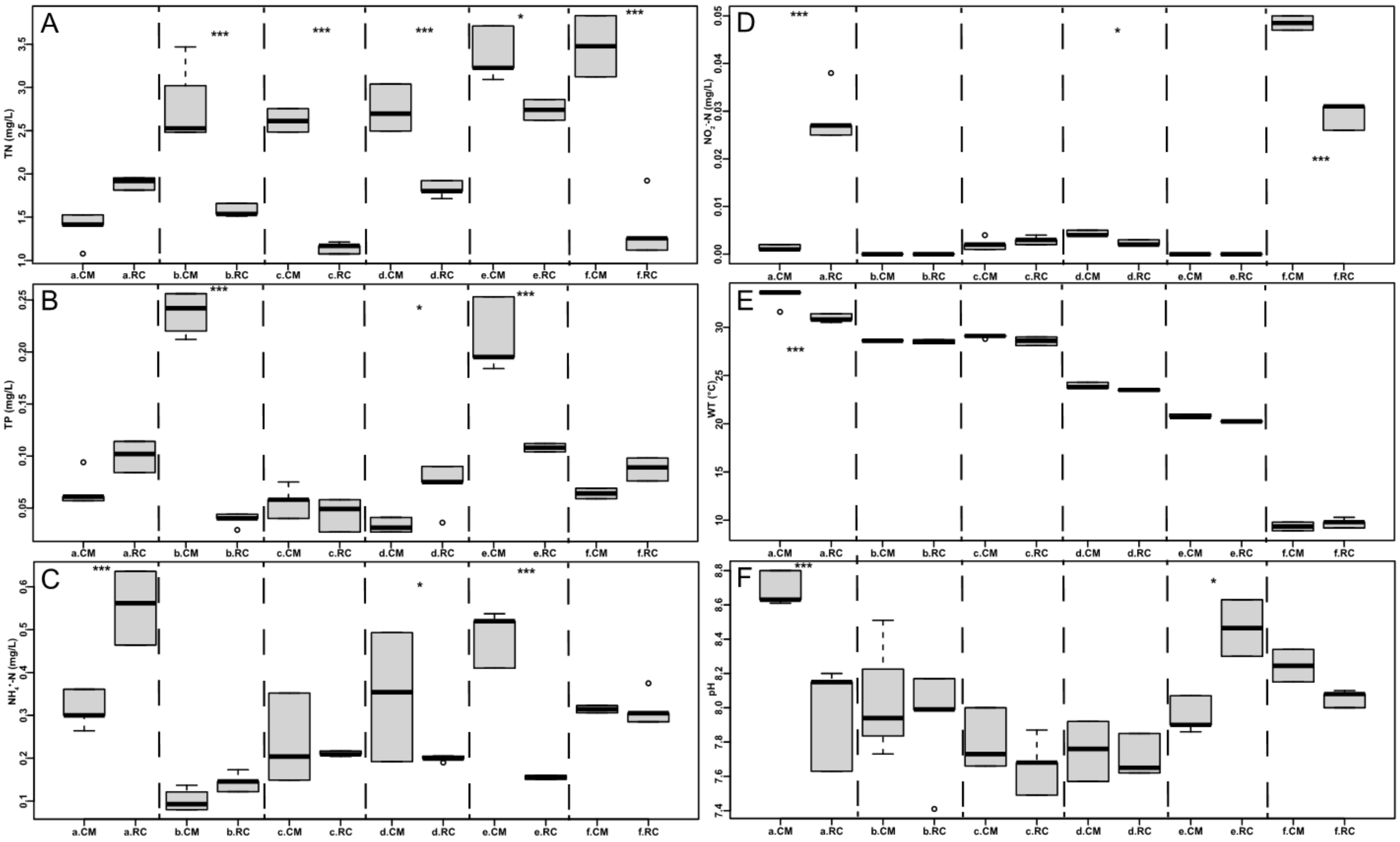

Fig S7. Physicochemical features of water samples between the RC and CM models. A, total nitrogen (TN); B, total phosphorus (TP); C, ammonia (NH_4^+-N); D, nitrite (NO_2^--N); E, water temperature (WT). F, pH. *, $P < 0.05$, **, $P < 0.01$, ***, $P < 0.001$. P-value was generated using paired ANOVA test and Tukey HSD method; center value represents the median of each physicochemical feature.

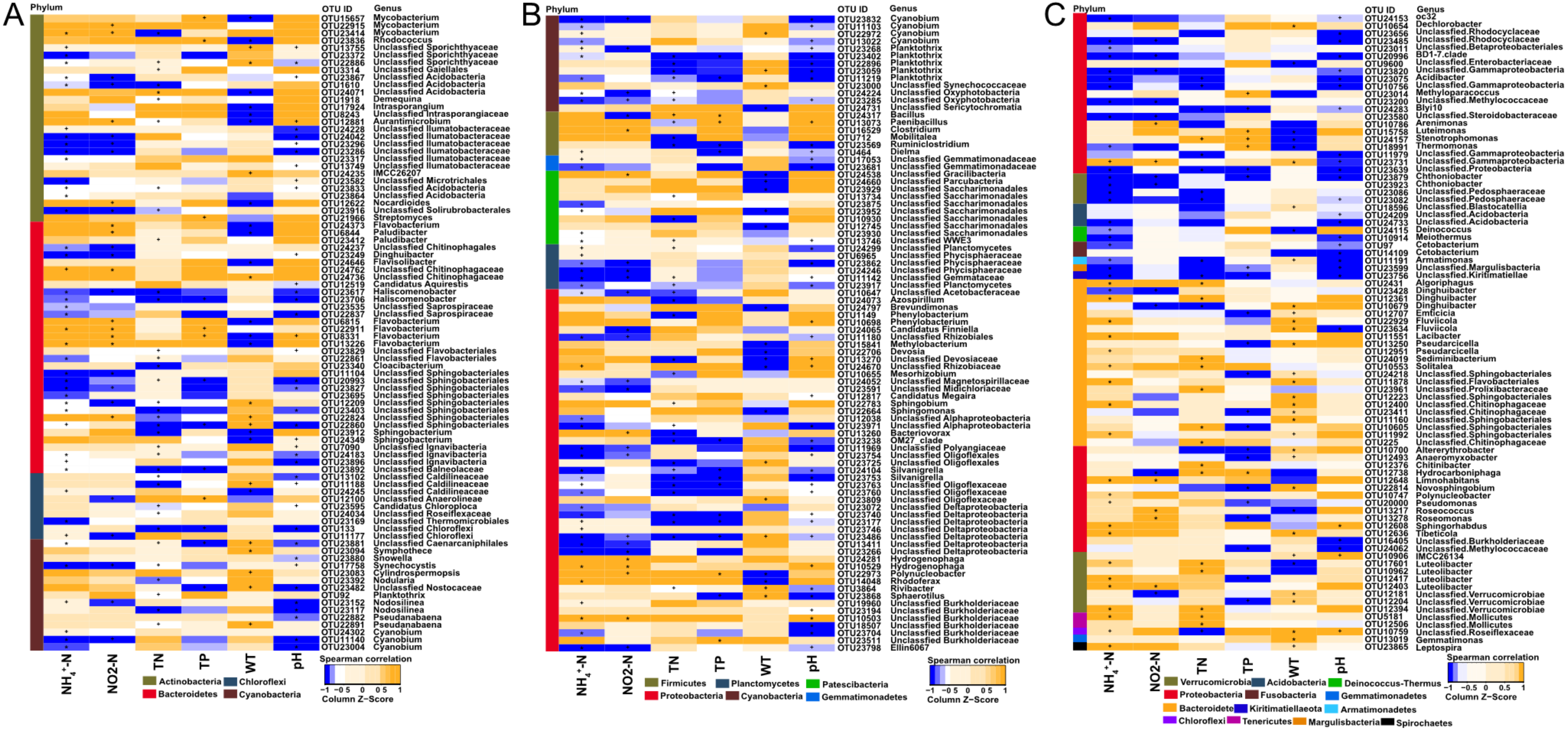

Fig S8. The correlation between physicochemical features and microbiome of water. Orange denotes positive Spearman correlation, while blue denotes negative Spearman correlation. The asterisk denotes $P < 0.01$; the plus sign denotes $P < 0.05$. TN, total nitrogen; TP, total phosphorus.

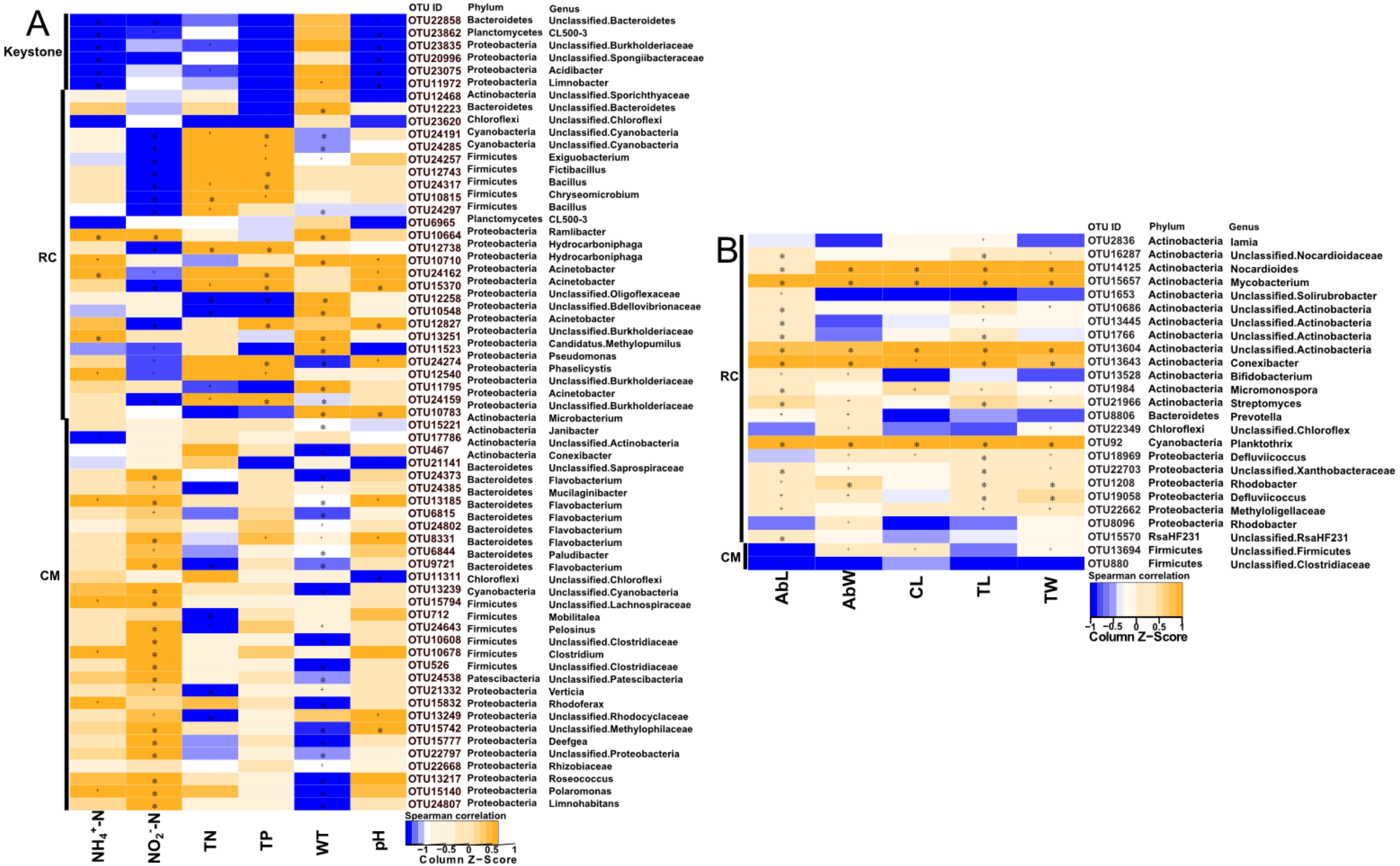

Fig S9. The association of physicochemical features of water with keystone or indicator OTUs of water microbiome (A), and the correlation of phenotypes of crayfishes with keystone or indicator OTUs of gut microbiome (B). Orange denotes positive Spearman correlation, while blue denotes negative Spearman correlation. The asterisk denotes P < 0.01; the plus sign denotes P < 0.05. TN, total nitrogen; TP, total phosphorus. TL, total length; TW, total weight; AbL, abdomen length; AbW, abdomen weight; CL, carapace length.
